# Raising concerns on questionable ethics approvals – a case study of 456 trials from the Institut Hospitalo-Universitaire Méditerranée Infection

**DOI:** 10.1186/s41073-023-00134-4

**Published:** 2023-08-03

**Authors:** Fabrice Frank, Nans Florens, Gideon Meyerowitz-katz, Jérôme Barriere, Éric Billy, Véronique Saada, Alexander Samuel, Jacques Robert, Lonni Besançon

**Affiliations:** 1 Independent researcher, Essaouira, Morocco; 2 Department of Nephrology, Hôpitaux Universitaires de Strasbourg, Université de Strasbourg, Strasbourg, France; 3 School of Health and Society, University of Wollongong, Wollongong, Australia; 4 Medical Oncology Department, Polyclinique Saint-Jean, Cagnes-sur-Mer, France; 5 Independent researcher, Strasbourg, France; 6 Biopathology department, Gustave Roussy Anti-Cancer Center, Villejuif, France; 7 Independent researcher, Nice, France; 8 Université de Bordeaux, INSERM Unité 1312, Bordeaux, France; 9 Media and Information Technology, Linköping University, Norrköping, Sweden

**Keywords:** Clinical research, Ethics, Scientific publications, Scientific publishing, IRB (Institutional Review Board)

## Abstract

**Background:**

The practice of clinical research is strictly regulated by law. During submission and review processes, compliance of such research with the laws enforced in the country where it was conducted is not always correctly filled in by the authors or verified by the editors. Here, we report a case of a single institution for which one may find hundreds of publications with seemingly relevant ethical concerns, along with 10 months of follow-up through contacts with the editors of these articles. We thus argue for a stricter control of ethical authorization by scientific editors and we call on publishers to cooperate to this end.

**Methods:**

We present an investigation of the ethics and legal aspects of 456 studies published by the IHU-MI (Institut Hospitalo-Universitaire Méditerranée Infection) in Marseille, France.

**Results:**

We identified a wide range of issues with the stated research authorization and ethics of the published studies with respect to the Institutional Review Board and the approval presented. Among the studies investigated, 248 were conducted with the same ethics approval number, even though the subjects, samples, and countries of investigation were different. Thirty-nine (39) did not even contain a reference to the ethics approval number while they present research on human beings. We thus contacted the journals that published these articles and provide their responses to our concerns. It should be noted that, since our investigation and reporting to journals, PLOS has issued expressions of concerns for several publications we analyze here.

**Conclusion:**

This case presents an investigation of the veracity of ethical approval, and more than 10 months of follow-up by independent researchers. We call for stricter control and cooperation in handling of these cases, including editorial requirement to upload ethical approval documents, guidelines from COPE to address such ethical concerns, and transparent editorial policies and timelines to answer such concerns. All supplementary materials are available.

**Supplementary Information:**

The online version contains supplementary material available at 10.1186/s41073-023-00134-4.

## Background

There are over 27 million scientific articles listed on the National Health Institute Platform PubMed. Previous investigations have shown that about 2% of scientists admitted to have fabricated, falsified or modified data or results at least once [1]. The business of scientific publication has surged during the last decade, including a staggering growth in the number of articles submitted and finally accepted for publication [2]. The peer review process is crucial for assessing the quality of hypotheses, methods, reliability of the data, and identifying any obvious ethical shortcomings. The Covid-19 pandemic was a stress test for the academic publishing system and unveiled several failures in processes evaluating quality of scientific publications [3–7]. Neglected or non-existent review procedures [3, 6], editorial conflicts of interests combined with expeditive peer review [3], inconsistent publications with, e.g., missing data [8], failure to retract or delayed retractions [6, 7], and irregularities in legal permissions are among the most common concerns seen in biomedical publications relating to the COVID-19 pandemic. The latter is particularly critical as it can directly put study participants at risk.

Regulation of clinical research became gradually an issue after the crimes perpetrated by the Nazis during World War II [9]. Later, these issues were reinforced by notoriously unethical studies conducted in the following decades, such as the Tuskegee Syphilis Study [10]. In the 1950 and 1960 s, the thalidomide adverse effects scandal reinforced this tendency by defining vulnerable groups such as, pregnant women [11]. More recently, ethics has evolved with new concerns for the protection of healthy volunteers following the TGN1412 disaster [12].

To prevent these ethical quandaries, countries have different governance system for ensuring appropriate moral conduct in clinical research. This commonly includes both legal requirements for conducting research, as well as guidelines for the oversight into and approval of ethical research. In France, this is done through the legislative framework. In this context, the French legislation was updated in 2016 with the Jardé Law on good practices in clinical research [13]. It should be noted that the legislative framework encountered in France is not the same as in other countries which may have other governance instead. French regulation requires that any experimentation on human beings must be approved by an independent ethics committee and depending on the complexity of the protocol, additional authorizations are required, especially regarding the collection of body fluids such as stool, vaginal secretions or urine.

In this paper, we present an investigation into papers published by the Institut Hospitalo-Universitaire Méditerranée Infection (IHU-MI ), a large clinical and research center in the city of Marseilles in the south of France. The IHU-MI employs over 700 people, covering a range of research topics related to infectious disease including basic biomedical research, epidemiological work, clinical trials,

This center has been the subject of research controversy since the advent of the COVID-19 pandemic, largely due to the actions of Professor Didier Raoult, former head of the institute. The original notoriety of the IHU-MI came from the now-infamous promotion of hydroxychloroquine, an anti-malarial medication, in combination with azithromycin for the treatment of COVID-19 [14]. This regimen was promoted as the most effective treatment for COVID-19 based on the results of a small, poorly-controlled observational study that has been described as having “major methodological shortcomings which make it nearly if not completely uninformative” and “fully irresponsible” in an independent review commissioned by the parent publishing company Elsevier [15–18]. This study also elicited negative peer-review comments on PubPeer, an independent review site that collates commentary on scientific studies, and from the French authorities [19]. The hydroxychloroquine/azithromycin regimen has remained popular in some minds despite increasingly robust evidence that it is ineffective in the treatment of COVID-19 and furthermore may increase the risk of death [18], demonstrating once more the danger of problematic and potentially unethical research [20]. Of note, IHU-MI has had previous retractions for alleged data fabrication, but thus far not due to ethical concerns [20].

Concerns on ethical approvals from IHU-MI have been raised outside scientific journals [21]. In August 2021, Elisabeth Bik pointed out issues about ethical approvals including research on vulnerable populations like homeless study participants [22]. These initial reports prompted us to further investigate potential concerns about ethics in the published literature from the institute. We have found and report below about highly worrying data in IHU-MI publications.

For post-publication critiques, the Committee on Publication Ethics (COPE) recommends referring to the journal or publisher policy [23], depending on the availability of an editorial policy for the reported issue. In case editorial policy does not take post-publication critiques into account, this policy should be amended. We can thus hope that the critiques formulated here might be helpful for journals to improve their peer reviewing policies. This is the whole meaning of our approach.

## Methods

### Investigation

Due to a great deal of national interest following publicization of poor research practices at IHU-MI, the French government investigated the unit and then launched legal actions in early September 2022. This followed a damning report from IGAS (Inspection Générale des Affaires Sociales) on the ethics and conduct of research taking place at IHU-MI during the period of investigation [24]. The seriousness of the accusations reported, combined with previous reports of questionable conduct and publication ban [20], made us question whether current academic editorial processes could have caught such concerns regarding the legal framework implemented at IHU-MI when conducting clinical trials. This paper provides the results of a detailed review covering the work of researchers at IHU-MI, analyzing published ethical statements.

After noticing that some Institutional Review Board (IRB) identification numbers were identical in several publications from the IHU-MI, we started by screening studies on IRB numbers on “Google Scholar”. We found several repetitions, and we finally noticed that one of the IRB approval numbers (09–022) appeared in hundreds of publications while the publications’ topics and the patients involved in studies were significantly different. We then used “Google Scholar” to identify all occurrences of this approval number.

We then decided to further investigate the bibliography of this institute by screening PubPeer reports and analyzing them. We did not contact directly IHU-MI since their answer to the PubPeer posts showed they were already aware of the concerns reported here. Furthermore, cyber and legal harassment made any direct contact with this institute more difficult than it should have been [21, 25, 26]. We only recently contacted Prof. Didier Raoult, former head of IHU-MI, since he was listed as editor-in-chief of the journals where some of the articles we report here were published but received no answer to this day. This list has also been reported to French Health Authority, namely the “Agence Nationale de Sécurité du Médicament” (ANSM), in charge of the evaluation of the legality of clinical studies, in July 2022. These publications were only evaluated regarding ethics criteria and our findings have no significance regarding the validity of their content.

We attempted to conduct a relatively systematic review of all recent biomedical research published by key authors at the IHU-MI, using “Google Scholar” profiles and PubPeer. This involved searching the bibliographies of senior scholars (i.e., Professors) at the institute, however given the sheer number of studies, we limited the search parameters to those published in the last 25 years. Screening on PubPeer was conducted by searching author names, and reviewing the resulting comments on papers. The results from PubPeer were then used to complete the data obtained from analysis of “Google Scholar” results. Frequently-repeating IRB-approval numbers were entered into “Google Scholar” to list of all their occurrences.

### Request for the official IRB approval

We qualitatively determined whether IRB approval was likely to have been granted for different studies. This was done by discussion among the authors, and we present the tabulated findings in the results of the number of separate published works that use the same IRB approval number.

### Contact of editors

While papers have been retracted in the past for issues concerning ethical approval [27], most of the Retraction Watch database entries seem to contain only Expressions of Concern (EOCs) or retractions relating to “ethical violations by author” or “lack of IRB/IACUC approval.” As such, it is not surprising that COPE did not provide guidelines on reporting and investigating IRB approval duplications such as what we have found. Indeed, current COPE guidelines on issues on ethics approval only focus on handling concerns at the time of submission of a manuscript [28] and not after publication or for cases of potentially fraudulent duplication of IRB approval numbers. As a result, we have further analysed the dataset to append the journal Editor-in-Chief’s name and email after screening the journals websites for information. In some cases, such as discontinued journals, the last known editor-in-chief was contacted. When no contact form or mail was available on the journal’s page, we contacted the editor-in-chief directly through academic mail found on the university affiliation’s website.

We thus contacted all editors of journals that published papers for which our analysis could raise concerns. The number of editors we contacted may seem very low compared to the number of publications we report, but many of them have been published in the same journal: for example “New Microbes and New Infections” published 135 of the studies we report. The low number of answers (despite reminder emails) led us to contact some publishers including Elsevier, for “New Microbes and New Infections”, without any satisfactory answer to this day. It should be noted that some of the authors of the papers we investigated are in many cases on the editorial team of the journals which have published the papers. Such concerns had already been highlighted with respect to this institute and some of their COVID-19 papers which had been peer-reviewed under 24 h in journals in which the authors presented with editorial conflicts of interest [3]. Related to our concerns, the journal “New Microbes and New Infections” is famous for being closely related to the team whose work we investigated [29] and had many members of the IHU-MI on the editorial board [30]. As such, editorial contact to report on the issues we have identified was indeed difficult.

At the time of writing, we have not approached the STM integrity hub. We have only contacted the ethics officer at Elsevier, since this publisher counts for most of the articles we report; from some answers of the editor-in-chief, we have no doubt that they did not intend to take any action.

## Results

### Investigation

**Fig. 1:**
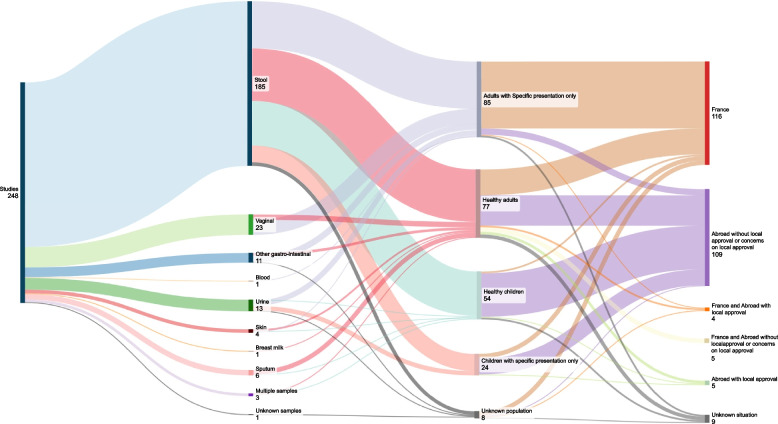
Various subjects, samples, and countries for the 248 studies with the IRB number 09–022

After cleaning, we noticed that the IRB approval number 09–022 had been used 248 times over 12 years (between 2009 and 2021). Reusing approvals is allowed if results are from samples originally approved by the committee and in compliance with local laws related to clinical research. However, we found that those 248 publications covered a large variety of samples (stool, vaginal secretions, urine, samples taken during surgical procedures), a wide array of populations (adults, children, healthy volunteers, obese patients, etc.) and countries (France, Senegal, Niger, Gabon, Saudi Arabia, etc.) as depicted in Fig. 1 (see the Additional file 2 “Table S1 – Studies_with_09–022_IRB.csv”). Among the 248 studies identified, we have found at least one that was conducted after the Jardé Law was implemented, as well as many more published after 2016 with no dates of patient enrollment identifiable.

Further investigation in the bibliography from IHU-MI showed a total of 456 studies that could have ethical and legal concerns of the same type: multiple and different studies with the same IRB, absence of legal authorization, recruitment starting before authorization was obtained, etc. (see Additional file 2 Table S2 “Table S2-Clinical_Research_Papers_With_Ethical_Concerns.csv”).

In biomedical research, researchers, authors, sponsors, editors and publishers all have ethical obligations with regard to the publication and dissemination of the results of research. Most, if not all, scientific publishing companies have subscribed to the declaration of Helsinki [31–34], which is also recommended by COPE [35].

#### Request for the official IRB approval

We could not access the original file with the IRB number 09–022, even after requesting this document from French authorities. However, we obtained a copy of the outline of the document (see Additional file 3). Based on our analysis, this form does not allow such a wide variety of samples, clinical conditions, and geographical origin to be documented. We could not find any reasonable explanation for such a multiplicity of identical occurrences in the literature. The original document should mention all those samples, conditions, and countries. If amendments have been made, they have not been explicitly mentioned in the articles from IHU-MI cited herein.

#### Contact of the editors

**Fig. 2:**
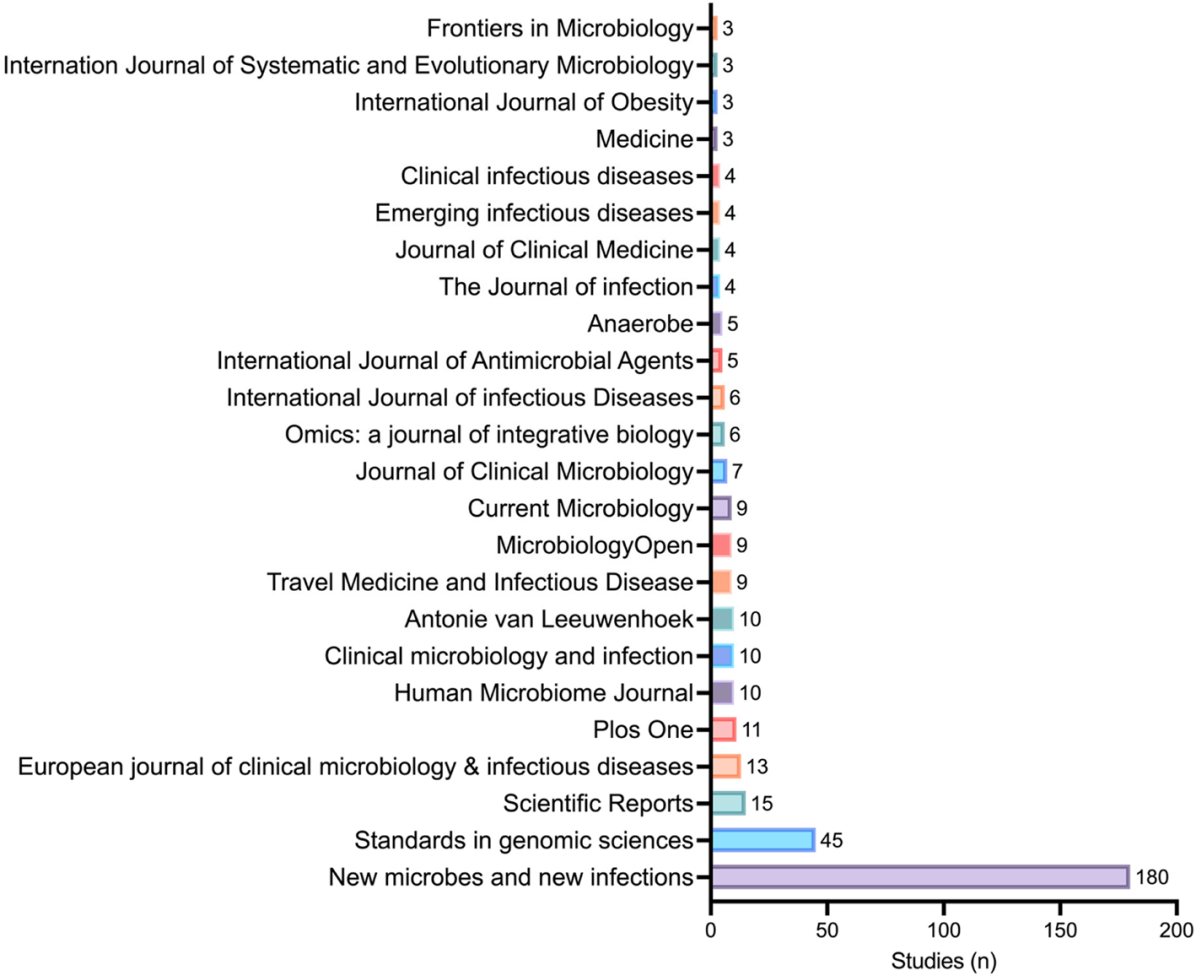
Journals involved in the 456 studies with legal authorization concerns

An overview of the journals that have published most of these articles is available in Fig. 2.

Out of the 85 journals that we contacted, 19 editorial teams have responded to our email while 66 have not replied to us yet. A complete list of the journals, dates at which they have been contacted, as well as date and summary of the response is available in Additional file 2 (“Table S3 – Editors contact.csv”).

## Discussion

In summary, among the studies we have investigated, 248 were conducted with the same ethics approval number, even though the subjects, samples, and countries of investigation were different. Thirty-nine (39) of the manuscripts we considered did not even contain a reference to the ethics approval number although they contained research on human beings. We have contacted the 85 journals that published these articles and only 19 of them have replied to our queries so far. However, a publisher, PLOS [36], has issued expressions of concerns for several publications we have analyzed over ethical concerns.

Taking French legal framework into account, we made a rapid analysis and this showed these studies could include different categories of research involving human beings (RIPH) i.e. 6 RIPH 1, 67 RIPH 2 and 202 RIPH 3 for which no legally-required authorization has been reported by the authors.

Since we have reached out to editors, PLOS Biology has issued a wide expression of concern for 49 articles published by this institute, including 12 for which we have reported concerns on ethics approval. Out of these 12 articles, ten use the same ethics approval number, namely 09–022. Given that we have not raised concerns about the remaining 37 articles, there appears to be some concern at the editorial level of the work done by IHU-MI more broadly than this investigation identified. Thus, our figures should serve as a baseline estimate of the total number of papers which may have issues that are associated with IHU-MI. To our best knowledge, no other public editorial decision has been made from other publishing venues yet, although we hope that the concerns from PLOS Biology will help either obtaining clarifications from the authors on such concerns, or drive other journals to publicly respond too.

Editorial practices in verifying ethics and lawfulness of clinical research are still very heterogeneous and all journal are not members of COPE. We wish to initiate a conversation to improve the ethical controls at the editorial level across published academic research, and for changes to facilitate post-hoc investigations in future work, despite not suggesting any mean of enforcing these controls.

While some publishers already require the upload of ethics approval, this is not a largely adopted requirement. We thus argue that the practice should become more widely and rigorously adopted, or that, at the very least, ethics approval numbers are provided as metadata along a submission such that post-hoc analysis could be done in a more systematically fashion through mining of submission’s metadata. This metadata could be made available through PubMed along with a basic description of the study and its targeted population, intervention(s), and country of study.

We argue that submission processes should be amended to require the potentially confidential upload of ethical documents linked to clinical research, and that editorial procedures should pay attention to the international (and potentially local) ethical framework for research by including, for instance, basic verification steps. This responsibility should absolutely not be placed on reviewers whose primary mission is to ensure the scientific robustness of the research as well as its relevance for publication. Indeed, much of this process could be easily automated by publishing companies such as Elsevier to avoid precisely the issues identified in this review. Placing the weight on an editorial responsibility would also facilitate further verification. Indeed, as we have ourselves experienced, independent researchers investigating the adequacy of ethical documents are not likely to obtain an answer from IRBs or ethical committees, while editors and publishers would have an easier and more legally anchored claim to request those documents. In conclusion, there is an urgent need for publishers to require clinical research approvals. This could be done by requesting validation from the sponsoring organization or from the authority that issued the IRB number. There is also a critical need for COPE to provide clear guidelines on how to report (for researchers) and how to handle (for editors and journals) issues with ethics approval in published manuscript. While we are aware that further editorial verifications could create additional and potentially difficult to navigate publication steps, we however believe that the recommendations we put forward here could easily be put in place without an increased bureaucratic cost. Indeed, our most drastic recommendation would be to normalize the upload of IRB documents for which IRB and publishers could easily implement regulations based on existing policies from journals which already implement such requirements.

We finally argue that editorial responses within a strict timeline should be put in place, such that journals and editorial teams have a responsibility to respond to ethical queries from researchers within a reasonable time [7] as well as disclose reasonable concerns that have been publicly raised on articles, and this even before reaching out to authors.

Since we cannot ask every editor to know every framework of every country for every type of clinical research, this would of course not solve all the problems, but we think that this might be a step forward to a better respect of ethics and thus of patient rights. While additional editorial constraints are unlikely to eliminate fraud or questionable practices altogether, they can help limit them, raise awareness about them, and facilitate their detection. We believe that the new editorial policies on ethics and IRB approval that we suggest would advance scientific processes in the same way that requiring the publication of clinical trials registration has facilitated research on the spin of medical research [37, 38], the prevalence of outcome switching, and other questionable reporting practices and likely help reduce these practices and adopt new standards to produce more robust and ethical research.

## Conclusion

We have presented a case study of 456 trials from the Institut Hospitalo-Universitaire Méditerranée Infection. Our investigation has revealed serious concerns on the ethics approvals of these trials ranging from the re-use of the same ethics approval number 248 times on trials with significantly different subjects, samples, and countries of investigation, to potential lack of local ethics approval for studies conducted abroad. To the best of our knowledge, our investigation is the first to reveal concerns over the potentially inappropriate reuse of ethics approval numbers on such a massive scale. While our concerns have been acted on by one publisher (PLoS), most publishers are either still investigating the issue or have not yet responded to us. This investigation thus highlights the needs for guidelines and processes for readers, reviewers, and editorial teams, to report and respond to ethics approval misuse and concerns.

## Supplementary Information

**Additional file 1: Figure 1.** Various subjects, samples, and countries for the 248 studieswith the IRB number 09-022. **Figure 2.** Journals involved in the 444 studies with legal authorization concerns.

**Additional file 2: Table S1.** Studies_with_09–022_IRB. **Table S2.** Clinical_Research_Papers_With_Ethical_Concerns. **Table S3.** Editors contact.

**Additional file 3:** Outline of IRB approval.

## Data Availability

The datasets supporting this article are available.

## Abbreviations

IHU: Institut Hospitalo-Universitaire.
IHU-MI: IHU Méditerannée Infection.
IRB: Institutional Review Board.
HCQ: Hydroxychloroquine.
ANSM: Agence Nationale de Sécurité du Médicament.
RIPH: Recherche impliquant la personne humaine i.e. research involving human subjects.
