# Supplementary material for: Raising concerns on questionable ethics approvals – a case study of 456 trials from the Institut Hospitalo-Universitaire Méditerranée Infection
Source: Res Integr Peer Rev. 2023 Aug 3;8:9. doi: 10.1186/s41073-023-00134-4 (PMC10398994; doi:10.1186/s41073-023-00134-4)
Supplement: Supplementary file 1 — Additional file 1: Figure 1. Various subjects, samples, and countries for the 248 studieswith the IRB number 09-022. Figure 2. Journals involved in the 444 studies with legal authorization concerns. [file 41073_2023_134_MOESM1_ESM.docx]

Supplementary Materials :

Figure 1 p2

Figure 2 p3


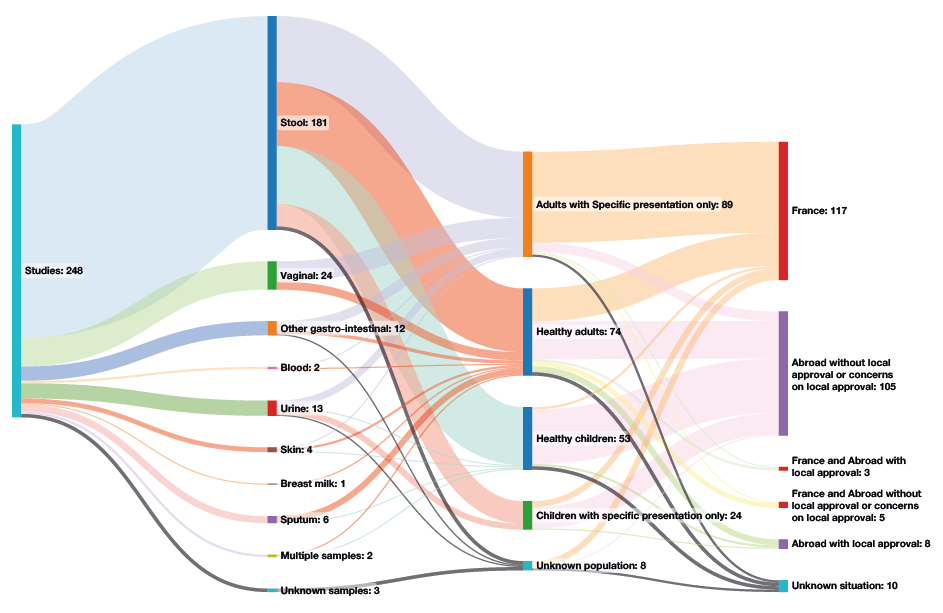


Figure 1: Various subjects, samples, and countries for the 248 studies with the IRB number 09-022.


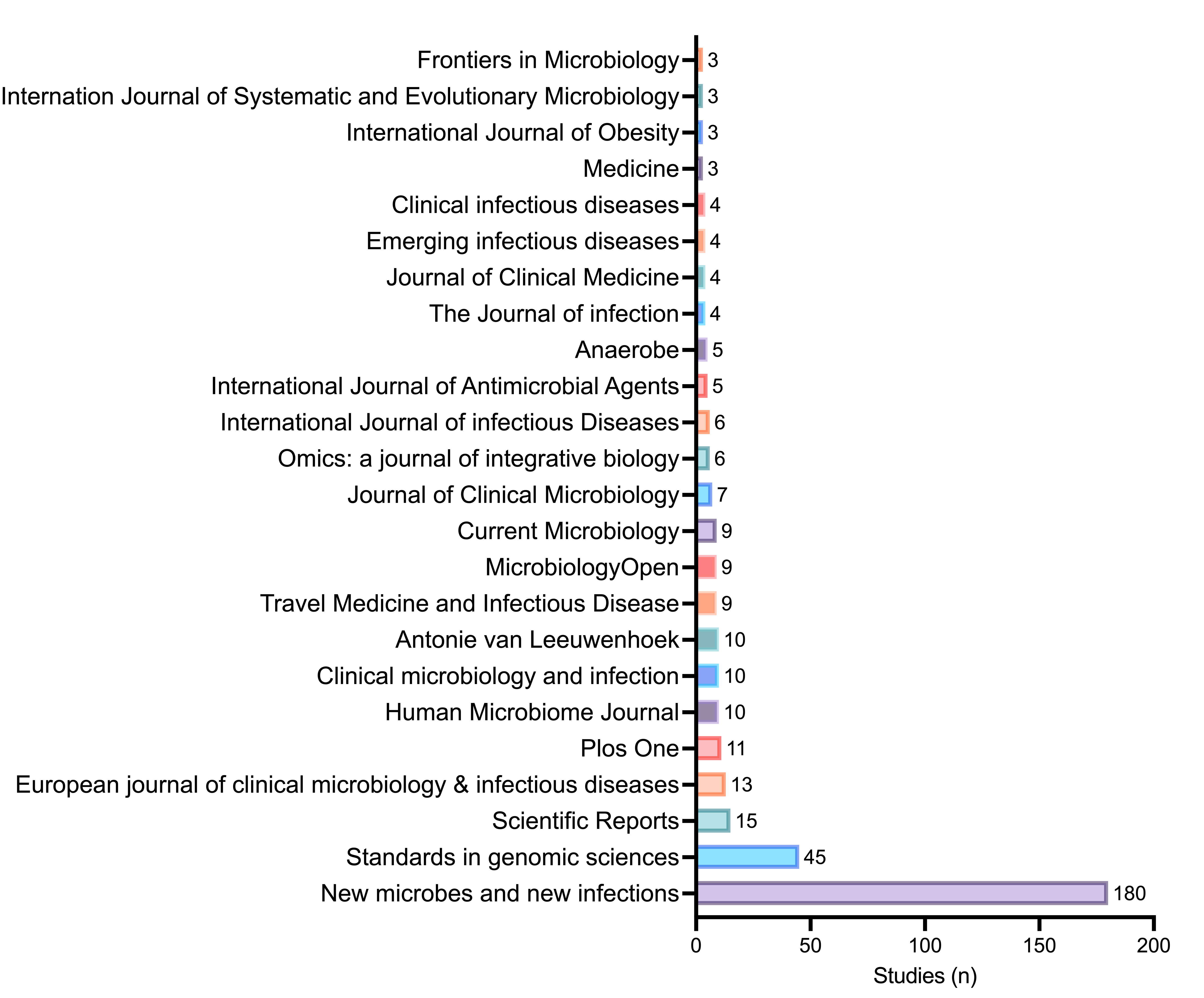


Figure 2. Journals involved in the 444 studies with legal authorization concerns.
