# Supplementary material for: Raising concerns on questionable ethics approvals – a case study of 456 trials from the Institut Hospitalo-Universitaire Méditerranée Infection
Source: Res Integr Peer Rev. 2023 Aug 3;8:9. doi: 10.1186/s41073-023-00134-4 (PMC10398994; doi:10.1186/s41073-023-00134-4)
Supplement: Supplementary file 3 — Additional file 3: Outline of IRB approval. [file 41073_2023_134_MOESM3_ESM.pdf]

COMITE D'ETHIQUE
DE L'IHU MEDITERRANEE-INFECTION

Pr [redacted]
Pr
Mr
Pr
Pr
Dr

N° : 2020-001

1) Nom de l'investigateur principal
[redacted]

2) Titre de l'étude
Déterminants des comportements d'automédication en médecine générale

3) Objectif(s) de l'étude

☐ Diagnostique ■ Epidémiologique ☐ Thérapeutique ☐ Physiopathologique

4) Moyens utilisés

☐ Animaux
Type ………………………………….. Nombre ………………………….
☐ Avis du Comité d'Ethique animale recueilli

☐ Patients
☐ Prélèvements cliniques ☐ Souches bactériennes ☐ Données médicales
■ Patients anonymisés ■ Information des patients (document fourni)
Type de prélèvements utilisés :

Nombre de prélèvements : Origine des prélèvements :
Des prélèvements ont-ils été réalisés spécifiquement pour cette étude ? ☐ oui ■ non

5) Publication(s) envisagée(s)
Oui

Je soussigné (investigateur principal) certifie sur l'honneur l'exactitude des données sus-mentionnées.

Signature : [redacted]

☒ Avis favorable du Comité d'Ethique
☐ Avis favorable sous réserve: ……………………………………………………………………
☐ Avis défavorable

Date: 29/01/2020 **Le Président**

[redacted]
